# Mapping experiences and perspectives of equity in international health collaborations: a scoping review

**DOI:** 10.1186/s12939-020-01350-w

**Published:** 2021-01-09

**Authors:** Marlyn C. Faure, Nchangwi S. Munung, Ntobeko A. B. Ntusi, Bridget Pratt, Jantina de Vries

**Affiliations:** 1 Department of Medicine, Faculty of Health Sciences, University of Cape Town, Cape Town, South Africa; 2 Division of Human Genetics, Faculty of Health Sciences, University of Cape Town, Cape Town, South Africa; 3 Centre for Health Equity, School of Population and Global Health, The University of Melbourne, Melbourne, Australia

**Keywords:** Equity, International health collaborations, Global health, Scoping review

## Abstract

**Background:**

Whilst global health research often involves international collaborations, achieving or promoting equity within collaborations remains a key challenge, despite established conceptual approaches and the development of frameworks and guidelines to promote equity. There have also been several empirical studies documenting researchers’ experiences of inequity and views on what is required to advance equity in global health collaborations. While these empirical studies provide critical insights, there has been no attempt to systematically synthetize what constitutes equity and how it can be achieved. To address this gap, we conducted a scoping review of qualitative studies, opinion and editorial pieces about what equity is and how it can be promoted in international collaborations.

**Methods:**

We conducted a scoping review to explore domains of equity in international health collaborations. This review included qualitative studies and opinion pieces or editorial pieces on equity in international health collaborations. We mapped the data and identified common themes using a thematic analysis approach.

**Results:**

This initial search retrieved a total of 7611 papers after removing duplicates. A total of 11 papers were included in this review, 10 empirical studies and 1 editorial piece. We conducted our search between October – November 2019. We identified 10 key domains which are important for promoting equity in international collaborations: funding; capacity building; authorship; sample ownership and export; trust; research agreement; acknowledging inequality; recognition and communication.

**Discussion:**

Our findings suggest that for international collaborations to be considered more equitable, it must at least consider the 10 domains we highlighted. The 10 domains map onto five key aspects of social justice theory, namely avoiding unequal power relations like subordination, group recognition and affirmation, promoting the well-being of all, inclusion in decision-making and ensuring self-development.

## Background

International health collaborations have been steadily increasing since the 1990s [1], often bringing together stakeholders from high-income countries (HICs) situated in the global North, where most funding sources are located, with stakeholders from lower- and middle-income countries (LMICs) located primarily in the global South. Whilst these collaborations may tackle a range of research questions, they often include questions about conditions that primarily affect people living in the global South [2, 3]. Driving this growth in collaborations are expectations that such collaborations will play a significant role in mitigating global health disparities [4–9]. Another driving factor is a “desire to be socially responsible” [10]. International collaboration in health research may have the effect of increasing clinical and research capacity in global South contexts and afford scientists in resource-poorer countries an opportunity to participate in or lead innovative scientific research and to publish [7, 11, 12].

Notwithstanding these potential benefits and driving factors, a range of critiques has been levelled against international health collaborations. Such collaborative arrangements have been accused of being exploitative of Southern researchers and communities, with some researchers labelling such practices as neo-colonial [13–16]. And while many Northern researchers may not set out to reproduce inequalities based on a colonial past, often collaborations have been seen as paternalistic, creating what Okeke has called, “the little brother effect” [17].

To address concerns related to international collaborations re-entrenching unequal relations, several bodies of literature have provided guidelines, imperatives and suggestions for equity in international collaboration. These include substantial conceptual accounts about what equity is, why it is imperative, and how it can be achieved [18, 19]. They draw on rich literature from political philosophy that explores the concept of equity and social justice and identify several components, including avoiding unfair power relations, recognition, inclusion in decision-making, and rights to self-development and adequate levels of well-being. Social justice means reducing unequal power relations such as subordination, exploitation, exclusion and violence [20–22] and includes recognition and affirmation of group difference [21, 23–25]. Three core aspects of misrecognition are: 1) cultural domination, 2) de-valuing and stereotyping social groups, and 3) rendering their knowledge and perspectives invisible [21, 26–28]. Accordingly, recognition encompasses affirmation of group difference, rendering the invisible visible and demonstrating respect. Social justice also means ensuring individuals and social groups, including those considered disadvantaged or marginalized, are included in making decisions that have a significant impact on their well-being [21, 29, 30]. Efforts should also be made to ensure that dominant power hierarchies are not reinforced, and those considered disadvantaged or marginalized are not included as tokens. Scholars often equate fairness with *consensual* and *deliberative* decision-making [21, 31–33]. Social justice further calls for ensuring self-development (understood as developing and exercising one’s capacities) and human flourishing (understood as achieving an adequate level of well-being for all) [21, 29, 34–36]. Philosophers argue for giving some priority to bringing disadvantaged or marginalized individuals and groups/communities up to an adequate level of well-being [34, 37, 38].

Ethics researchers have applied these and other concepts from the philosophy literature on health and social justice to explore what equity means for international research [39–42]. This conceptual work has proposed that international research collaborations should generate new knowledge to improve the health and well-being of LMIC populations, particularly those considered disadvantaged or marginalized; foster their and LMIC researchers’ meaningful participation in decision-making about its conduct; and build research capacity in LMICs [39, 40, 43]. However, debates continue as to whether international research collaborations even have a responsibility to contribute to justice beyond the micro-level (i.e. achieving a fair balance of burdens and benefits during individual projects). Some scholars have argued that international research collaborations should not be expected to contribute to reducing global health disparities and building research capacity, whereas others strongly disagree [43–45].

Additionally, several guidelines and frameworks have been developed that seek to promote more equitable collaborations. These include the “Responsible Conduct in the Global Research Enterprise” [46], the Montreal Statement on Research Integrity in Cross-Boundary Research Collaborations [47], the guidelines developed by the Commission for Research Partnership with Developing Countries [48] and the COHRED Fairness Index for international collaborative partnerships [49]. Moreover, there have been several qualitative studies reporting on researcher’s experiences within international collaborations [4, 50].

While these papers provide necessary empirical evidence for what constitutes both inequity and equity, there has been no systematic attempt to synthesize empirical studies in this domain. The absence of such a synthesized understanding challenges researchers’ ability to pro-actively engage in and understand how equity in research collaboration should be promoted. To address this gap, we conducted a scoping review, mapping and synthesizing research from qualitative studies investigating dimensions of equity in international health collaborations.

### Aim

To develop, through synthesizing evidence from published articles, key areas considered critical to fostering equity in collaborations identified by stakeholders involved in international collaborations.

## Method

Scoping reviews are generally used to map emerging evidence relating to broad topics [51–53] and could focus on: synthesizing the available evidence in a field; defining and clarifying concepts and ideas; identifying where more information is required or identifying a question for a systematic review [51]. For this review, we were interested in synthesizing evidence relating to equity and developing greater clarity on various dimensions considered important for international collaborations to be equitable.

### Identifying relevant data

#### Study selection

The third step of the framework involves developing and applying criteria to consistently and transparently select relevant studies. In this review, we applied the following criteria: studies which reported on a researcher’s or scientist’s perspectives or opinions on what they perceived made an international scientific collaboration (in) equitable, or studies which reported on empirical qualitative evidence about what made international scientific collaboration (in)equitable. Studies which reported on tools, frameworks, guidelines or regulations that could be implemented to ensure equity were excluded. Studies reporting on any normative or philosophical accounts of what constituted equity in international research collaborations were also excluded (Table 1).

**Table 1:** Table of search terms based on guidelines by Peters et al. [79]. For full search strategy adapted for each database, please see Appendix 1

Population	Research Personnel [MeSH] OR Researcher OR researchers OR investigators OR scientists
Concept	Ethics OR ethical OR equity OR equitable OR equality OR fair OR fairness OR values OR justice OR “social justice”
Context	International Cooperation [MeSH] OR Cooperative Behavior [MeSH] OR Collaboration OR collaborators OR cooperation OR international collaboration OR decision making OR international scientific collaboration OR international research collaboration [AND] “Empirical studies” OR “qualitative research” OR “qualitative methods” OR opinion OR perspective

Two reviewers (MCF and NSM) applied the selection criteria, first at the title and abstract level and subsequently at the level of full-text articles. Each reviewer independently reviewed articles at the title/abstract and full-text stages. At the title/abstract review stage, articles, where both reviewers agreed, were included in the full-text search. Where there was a discrepancy, a third reviewer (JDV) adjudicated, and decided if papers should be included for full-text review. For the full-text search, articles where both reviewers (MCF and NSM) agreed, full-text articles were included. A third reviewer (JDV) applied the selection criteria to all full-text manuscripts where the two reviewers (MCF AND NSM) disagreed or were unsure.

#### Inclusion criteria

For empirical studies to be included in this review, they had to meet the following criteria:

- qualitative studies
- study participants were researchers or those involved in research
- investigated experiences, ideas, concepts, values related to equity within international collaborations.

For opinion or editorial pieces, the articles had to have a focus on equity within international research collaborations, particularly describing experiences, ideas, concepts and values relating to equity.

Articles which did not meet these criteria were excluded from the review.

#### Mapping the data

Once studies were selected, the fourth step of the scoping review process is to map or “chart” the data [52]. While there are no commonly agreed methods for this step, what is important is that the method systematically organizes the data into relevant themes [52, 53] – not dissimilar to how conventional thematic analysis would operate in qualitative studies [54]. We approached this by firstly, extracting publication details such as author institutional affiliation and dates of publication. We then extracted data related to what studies reported as important for creating equitable international scientific collaborations. For this stage of the review, articles were read through several times and a thematic coding scheme was developed inductively by one reviewer (MCF) in discussion with another author (JDV). After themes were derived and papers coded using broad themes, sub-themes were developed. During each of stage of thematic development, themes and sub-themes were presented to two of members of the research team (NSM and JDV) who had also read through included papers and could provide feedback on the accuracy of themes.

## Results

### Search results

**Fig. 1:**
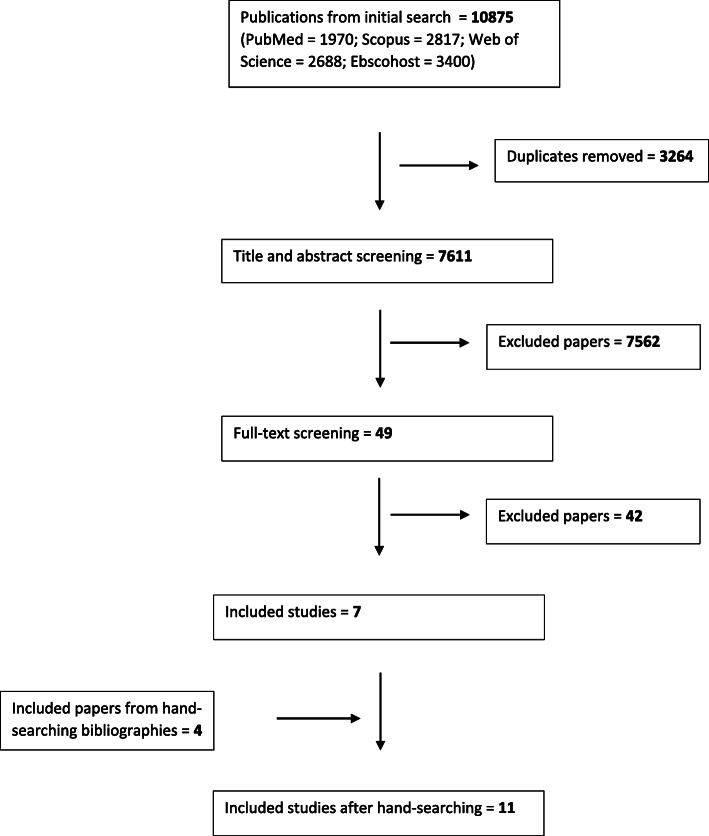
Strobe diagram showing flow of searches

**Fig. 2:**
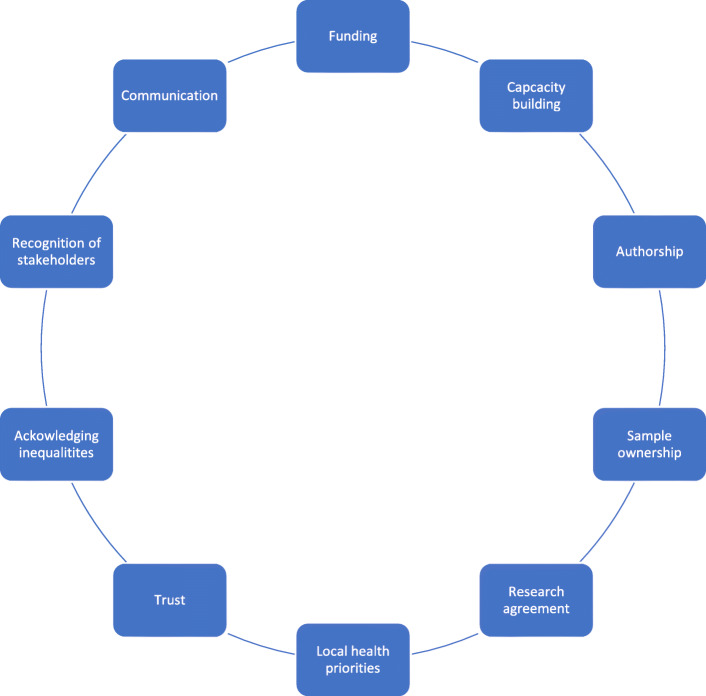
10 key areas which critical for developing equitable international collaborations

We retrieved a total of 7611 results after duplicates were removed. After screening all papers at the title and abstract level, 49 papers were selected for full-text screening. A total of seven papers met the inclusion criteria, with an additional four papers identified for inclusion after searching the bibliographies of the papers that matched the inclusion criteria. In addition to hand-searching, we also, through Google Scholar and the citation tracking function, checked for papers that had cited the papers included in our study but found none. A total of 11 articles were included in the final analysis, with 10 of these being qualitative studies, and one an editorial piece. (see Figs. 1 and 2).

### Overall findings

Most of the studies included in this review involved a range of participants in the research process, namely: bio-medical and social science researchers; university administrative staff and managers; community members; and government officials. Of the 10 empirical studies, three studies included participants from a single country in the global South. The remaining studies (*n* = 7) included participants based in the global North and South, with some participants being based in both contexts (see Tables 1 and 2 for a summary of demographics of included studies). All papers were published between 2003 and 2019.

**Table 2:** Summary of included studies

First Author and last authors	Year of publication	Title of paper	Study design	Number of interviews or FGDs	Kinds of participants	Location of study participants
B. Jentsch; C. Pilley	2003	Research relationships between the South and the North: Cinderella and the ugly sisters?	Case study analysis; interviews	2 case studies; 7 interviews	Researchers	United Kingdom; Bangladesh; Thailand
K.A. Muldoon; D.M. Moore	2012	Supporting Southern-led Research: Implications for North-South Research Partnerships	Interviews	12 interviews	Nurses; doctors; counsellors; information technology personnel; laboratory technicians; human resource managers	Uganda
P. Tindana; M. Parker	2014	Ethical issues in the export, storage and reuse of human biological samples in biomedical research: perspectives of key stakeholders in Ghana and Kenya	Interviews and focus group discussions	44 interviews; 6 focus groups	Researchers, fieldworkers; research assistants; laboratory staff; members of ethics committees; directors of research institutions; community representatives	Kenya and Ghana
F.M. Okwaro; P. W. Geissler	2015	In/dependent Collaborations: Perceptions and Experiences of African Scientists in Transnational HIV Research	Ethnography (in-depth interviews, focus group discussions, informal chats, and observations with scientists and other staff)	29 interviews; 6 focus groups	African scientists, laboratory technicians, mobilizers, administrators, nurses, and counsellors	East Africa
K. Moodley; S. Singh	2016	“It’s all about trust”: reflections of researchers on the complexity and controversy surrounding biobanking in South Africa	Interviews	21interviews	Researchers (bio-medical); biobanking and governance experts	South Africa
A. Walsh; E. Byrne	2016	“The way the country has been carved up by researchers”: ethics and power in North-south public health research	Interviews	53 interviews	Researchers (bio-medical and social science); government officials; NGOs	Zambia; and Northern researchers involved research based on Zambia
M. Parker; P. Kingori	2016	Good and Bad Research Collaborations: Researchers’ Views on Science and Ethics in Global Health Research	Interviews	22 interviews	Principal investigators; research funders; network coordinators; clinical trial managers; epidemiologists; laboratory managers; IT specialists, and statisticians, clinical researchers and managers of research institutions	South-East Asia, South Asia, East Africa, West Africa, Europe, Oceania, and North America
N.S. Munung; J de Vries	2017	Equity in international health research collaborations in Africa: Perceptions and expectations of African researchers	Interviews	17 interviews	Principal investigators; co-principal investigators; research	8 African countries
J. Guzman; E.R. Mendoza	2017	Ethical challenges for international collaborative research partnerships in the context of the Zika outbreak in the Dominican Republic: a qualitative case study	Interviews and focus group discussions	39 (includes both interviews and focus groups)	Researchers; NGOs; private organizations; government officials	Dominican Republic
T.F.L. Matenga; O. Mweemba	2019	Contemporary issues in North–south health research partnerships: perspectives of health research stakeholders in Zambia	Interviews	20 interviews	Principal investigators; project managers; laboratory managers; clinical researchers; academic researchers; members of ethics committees; government officials	Zambia
F. Binka	2005	North–South research collaborations: a move towards a true partnership?	Editorial	N/A	N/A	N/A

At the level of authorship, six of the 11 first authors were from LMICs, with six out of 10 senior authors being from HICs. See Table 3 for a summary of authorship demographics.

**Table 3:** Breakdown of first and last author by geographical origin

	No of articles	LMIC		HIC	
		First Author	Last author	First author	Last author
Empirical	**10**	5	4	5	6
Opinion	**1**	1			

After conducting a thematic analysis for the 11 included papers, 10 themes relating to equity in international scientific collaborations were identified: funding; capacity building; authorship; sample ownership and export; research agreement; meeting health priorities in Southern-based contexts; trust; acknowledging inequality; recognition of all stakeholders; and communication.

### Funding

Funding was a central determinant of equity and was reported on in 6 of the 11 articles included. Participants described that since research funding primarily originates from the global North, power is therefore located with the Northern partners [2, 55]. This arrangement has important implications for equity. For example, two studies reported that their participants, based in the global South, often had little influence on how and where money was spent [2, 56]. Another study reported that participants observed equitable collaborations when researchers based in LMICs can apply directly for funding and subsequently approach researchers based in HICs to collaborate [57]. Relating to dependency, participants in one study highlighted that researchers in the global South often struggle to identify calls for funding, and have insufficient capacity (particularly with regard to human resources) to develop funding proposals for international funding [58]. Another study noted that African researchers typically do not have access to sustainable funding which limits their ability to independently raise funds and pursue their own research agendas [3]. Participants lamented their own dependency on Northern funders since there was a serious lack of funding for research from local governments in the global South [2].

### Capacity building

A second key theme that emerged from our analysis was capacity building, which was identified in 10 out of 11 articles. For most studies, capacity building was defined as ensuring that the partner located in the global South received relevant training. Relevant training was considered training related to the current research project and which would aid in attracting independent research funding in the future [2, 3, 56–61]. Key types of capacity building described included grant writing skills, methodological and analytical skills and formal qualifications such as masters or doctoral degrees. One study envisaged capacity building to involve training researchers to use new technologies which would help them perform innovative research that would be internationally recognized [56]. In a different study, capacity building meant increasing infrastructural support [3]. Specifically, the articles emphasized that researchers based in the South should not simply be viewed as ‘sample collectors’.

Two studies were critical of traditional notions of capacity building, asserting for instance that the objective of capacity building activities should not simply be to upskill researchers but to ensure that over time, Southern researchers come to rely less on the technical assistance of Northern partners to conduct research [3, 59, 62]. Importantly, two studies cautioned that capacity building was not only a one-way process where researchers based in LMICs are educated by researchers based in HICs, but rather that capacity building should be a reciprocal process where both partners learn from each other since both partners are contributing different skills and resources to the project [55, 63]. Conceptualizing capacity building as a unidirectional activity risks obscuring the skills and knowledge of Southern researchers, while unwittingly propping up Northern researchers as intellectually or academically superior [63].

### Authorship in scientific collaborations

The third emerging theme we identified related to authorship. Five studies highlighted the importance of fair authorship practices [3, 55, 60, 61, 63]. The primary concern was that researchers based in LMICs are duly recognized – at a minimum, that they should be listed as co-authors, but ideally should be supported to take on first- or senior author roles. Such a change would involve empowering scientists from the South to lead analyses, initiate and manage the writing and publication processes.

### Sample ownership and export

Another theme related to sample ownership, export and secondary uses of the samples [2, 60, 64].. Participants in two of the 11 studies articulated concerns that when samples were exported from LMICs for analysis in laboratories in HICs, there is little control over the use of those resources and the knowledge generated from those samples [3, 57]. Of particular concern was that once samples were only stored abroad, it would be difficult to ensure that the researchers involved in the initial collection of these resources would continue to be involved or recognized in subsequent work or publications emanating out of the research [3, 64]. And this was considered to undermine equity in international research collaborations.

### Research agreements

The fifth theme we identified was the importance of clear research agreements to promote issues of equity. Four of the studies noted that for collaborations to be equitable, details of the collaboration, such as how funding will be spent, division of labour, decision-making arrangements and authorship policies, should be clearly articulated at the outset [3, 57, 58, 63]. Studies also underscored the critical role of researchers based in LMICs in negotiating fair terms for such agreements [56, 57, 59].

### Local priorities

An important criticism of international collaborations is that research priorities in the global South are not taken seriously by Northern collaborators. Three studies reported that international collaborations should consider or focus on local health priorities as key objectives of studies as opposed to researchers based in HICs deciding what those objectives should be [3, 55, 61]. Studies also described the importance of local communities benefitting from international collaborations. While the study did not articulate what these benefits could be, the article described how difficult it was for Southern-based researchers to spend money on research, but not being able to financially assist their research participants who were often struggling to survive [56].

### Trust

Trust was another key relational aspect of developing equitable partnerships. Most notable was the relationship between trust and funding. In one study, the Northern and Southern participants reported that funders tend not to trust Southern researchers to manage funds and preferentially trust Northern partners to handle fund management [55]. Another paper reported that as a result of funding generally being located in the North, Northern partners often dictate how money is spent, without having to account to Southern partners [2]. Such issues of mistrust could be bypassed (to some extent) when Southern staff were managed by Southern researchers and managers who had the cultural sensitivity necessary to create a cooperative working environment [63]. However, trust often takes a long time to build between collaborators, and having open and honest discussions between collaborators is essential for doing so [56]. Additionally, there were two factors which signalled a collaborator could be trusted. First, it was the reputation “and the absence of undesirable qualities” and second, this was also extended beyond the individual to include the trust funders placed in institutions where researchers located [56]. Yet trust at the inter-personal level is insufficient to mitigate the asymmetrical power relations between collaborators that are rooted in uneven international funding arrangements.

### Acknowledging inequality

Studies also reflected on the importance of acknowledging that most collaborations between researchers based in LMICs and HICs are inherently unequal, and it would be disingenuous to pretend that the inequality did not exist. For one study, this acknowledgement was about researchers from both the global North and South to acknowledge the different capacities each team brings to the collaboration and the limitations of their contributions [56]. Other studies reflected on the importance of acknowledging more material and structural inequalities that are inherent in international collaborations, such as differences in training, exposure to technology, skill sets, and funding and other resources, which is as a result of past and contemporary geopolitical arrangements [1, 2, 55, 58].

### Recognition of the contribution of all collaborators

Recognizing the role of various stakeholders contributing to the success of the project was also seen as critical for equity. Five of the included studies reported that their participants felt that recognizing skills, abilities, and local expertise was an essential element of reducing inequality. In practice, this also entailed acknowledging actors who would usually not qualify for co-authorship [2, 3, 55, 60, 63]. From some studies, this recognition also entailed broadening the group of people who get recognized and should include stakeholders such as those collecting samples and data [56]. In line with views on capacity building, one study emphasized the importance of recognizing the limitations of researchers based in HICs as well [63]. Related to this was the importance of valuing equality. The studies described that what often stifles more equitable relationships is when some roles are de-valued, or other roles receive relatively greater recognition or prominence [56, 63]. Such arrangements “undermine the opportunity for change when Northern personnel, as ‘capacity providers’ are unable to admit to need, and Southern researchers, as ‘receivers’, are not acknowledged for existing capacity” [63].

### Communication

Communication emerged as another component for creating equitable relationships. Specifically, three studies observed that collaborations should be set up in ways that allow for open and honest communication between researchers at the outset of the collaboration [1, 2, 61]. For example, one study noted that open communication at the outset allowed all stakeholders to feel heard and be taken seriously [56]. Another study noted that transparent communication at the start of the collaboration was critical to clarify roles and expectations, and would help prevent conflict at a later stage of the collaboration [2].

## Discussion

This scoping review synthetized empirical evidence about the experiences and understanding of equity in international research collaborations. Our review identified 10 dimensions of international scientific collaborations that were considered to be important in promoting equity. At the level of structural aspects of the collaboration, issues relating to where funding is held and control over funding, authorship arrangements, two-way capacity building, sample ownership and export were described as important in fostering equity. Also essential were fair research agreements negotiated at the outset of the collaboration, with LMIC researchers being able to ensure that study benefits are aligned to local health priorities. The review also highlighted more relational aspects of research collaborations that underpin equity such as explicitly acknowledging and discussing the impact of existing inequality, and ensuring recognition of the work of all stakeholders in the research endeavour – including the contributions for instance of the people organizing sample collection efforts. Moreover, trust was critical to fostering equity, alongside the need for researchers to be able to communicate openly and transparently. Structural and relational dimensions were interrelated in the sense that the articles revealed that to achieve equity with regards to a relational aspect, also requires attention to be given to a structural dimension. For example, in our reporting, we noted that while trust-building is a key relational dimension, it was often influenced or determined by funding arrangements, a structural element.

Beyond the structural and relational, we also draw attention to the fact that the identified domains map onto five components of social justice identified in the political philosophy literature. Funding location and control over funding issues are consistent with unfair power relations of subordination, where a privileged few get to determine the rules and make decisions that apply to many others [20]. Subordination encompasses an unfair division of labour in the workplace between those who plan and those who execute [21]. This has been a feature of HIC-LMIC international research collaborations [3, 63]. Acknowledging inequities, recognition, and authorship domains are consistent with social justice as recognition, which entails affirming group differences and valuing others, especially those who have been marginalized by social institutions and norms [21, 23, 24]. In the international research context, LMIC researchers have been marginalized by funding institutions and collaboration norms. Sample ownership issues were connected to unfair power relations (i.e., subordination or control by others) and recognition. Capacity building and trust speak to rights of self-development and achieving adequate well-being, specifically relational aspects of well-being: affiliation and commune. Commune refers to relations of harmony with others [65]. Where research collaborations are characterized by relations of discord (i.e. ill will, us versus them), they can negatively affect members’ well-being. Finally, open communication and research agreements that are characterized by fair terms and transparency are consistent with norms of fair decision-making and deliberation. Inherent in existing accounts of deliberative democracy are norms such as inclusion, reciprocity, reasonableness, and publicity [29, 31]. We thus propose that these five components of social justice – namely avoiding unequal power relations, group recognition and affirmation, inclusive decision-making, promoting everyone’s well-being and ensuring self-development – (at least) are relevant for equity in international collaborations. Future conceptual work should explore what upholding particular components of social justice means for the relevant domains of equity in international research collaborations identified in this paper, i.e. what reducing unfair power relations calls for in terms of funding. How each domain should be defined can draw on rich work from political philosophy.

In comparing our findings to the (limited) ethics literature that has applied theories of health and social justice to develop guidance on equity in international research collaborations, we note that relational matters such as recognition, communication, trust, and acknowledging inequities are largely not discussed. This paper’s findings, however, indicate that relational aspects of equity in international research collaborations are important, and we suggest they, therefore, require further consideration and exploration. Matters of building research capacity, inclusive decision-making throughout the research process, and ensuring collaborations are responsive to the health care and system needs of LMIC populations are discussed. However, the implications of inclusive decision-making for developing research agreements have not been specifically focused upon [39, 41, 66].

Fair funding arrangements was a key theme which emerged as critical in promoting more equitable collaborations. A key criticism of funding arrangements was that funding originates in the North, and Northern partners often have control over how and where money is spent, which severely restricts Southern researchers’ power in collaborations. Although there appeared to be a perception that funders tend to prefer Northern partners to oversee funding activities, it is not clear if this is indeed the case. For example, funding from national government agencies is more likely to require that the funds be held at an institution in that country, irrespective of where the research is carried out. This may differ in cases where the primary focus is research in LMICs. The dynamics of funding have recently started to be explored, though the issue of control identified in this paper does not always feature. For example, Pierson and Millum explore what individual health research funders are ethically obligated to do to help reduce global health disparities [67]. However, they are more focused on how funders should allocate their resources to various illnesses than to how resources should be allocated amongst researchers to reduce relations of subordination. Pratt and Hyder, in contrast, argue that equity means funders should incentivize LMIC ownership of international research agendas by restricting lead applicant eligibility to LMIC institutions [68]. An example of where that was the case is the H3Africa Consortium [69].

Moreover, with regards to structural elements, whilst the articles we reviewed, described the importance of capacity building and fair funding arrangements, these domains were considered almost exclusively between researchers or research groups. What is missing from almost all the papers included in this review are institutional forms of equity. For example, forms of capacity building described in the articles were training in research methods and new techniques, formal graduate training programmes and developing grant writing skills so that Southern-based researchers can attract their own funding. While such forms of capacity building are critical, they are only focussed on capacity building directly related to the research and only consider an individual or group. Only one study in this review reported on the importance of building more structural forms capacity, for instance, strengthening ethics review capacity [3]. The articles included in this review did not consider broader frameworks for building institutional or structural capacity [70, 71], or the importance of also building social science expertise [72].

Similarly, while the articles we included did report on the importance of funding to support research in lower- and middle-income countries, they narrowly focused on direct funding for research. Indirect costs of research funding are critical for institutional capacity building, such administrative and financial offices which provide critical support for research to flourish [73]. Importantly, the under-funding of institutional capacity, especially at the administrative level, has the potential to erode capacity of Southern researchers and institutions [73].

Finally, one of the critiques of international research collaborations is that researchers in LMICs often do not lead publications. Bibliometric studies on authorship in global health research reveal that a significant proportion of articles reporting on international health research conducted in LMICs had an author affiliated to the LMIC where the research was carried out [74, 75]. Overall, less than 15% of publications on global health research did not have a co-author from the LMIC of interest [75]. The problem seems to lie more with the authorship order, whereby authors from LMICs are less likely to be first or last authors [76, 77]. This scoping review highlights a desire for authorship parity in international health research collaborations.

### Implications

While the 10 dimensions we identified provide overall guidance about what necessary dimensions of equitable collaborations, the findings of this review also underline the highly complex nature of achieving equity in collaborations. This complexity relates, firstly, to the differing perspectives from researchers and other stakeholders about which domains are required to establish equitable relationships. Secondly, there is also a variety of perspectives about what each domain requires to achieve equity in collaboration. For example, capacity building often means different things to researchers depending on their context. While many themes are generally consistent across many studies, such variance presents important challenges to ensuring equity. What this scoping review perhaps suggests is that finding standard ethical arrangements across time and space is difficult, and these aspects should be worked out in different contexts and collaborations. While such variance is important, especially considering multiple contextual factors, having no baseline shared, and standardized understanding of equity may also potentially undermine equity within international collaborations [78]. While these dimensions cannot be standardized across all collaborations, our findings demonstrate these are the key areas which must be carefully considered for international collaborations to develop more equitable practices.

In addition, what also needs to be considered is that achieving equity often requires both structural and relational obligations to be fulfilled beyond individual researchers and/or institutions. For example, as it relates to matters of authorship or building trust, these could be worked out between researchers and institutions. However, funding practices are often determined by funders, their policies and the constituencies to whom they are accountable, well beyond the control of individual researchers and research institutions.

### Limitations

One important limitation of this study was the small number of papers included in the review. This could suggest that our search strategy did not yield adequate results. We developed a comprehensive search strategy that included as many relevant key terms as possible, searching in as many relevant databases as we had access to. The initial search yielded a large number of publications, yet surprisingly few papers were included. To expand our search, we hand-searched the bibliographies of all included studies for any papers that we may have missed in our original search, and we found an additional four papers which met our inclusion criteria. In addition to hand- searches, we also, through Google Scholar and the citation tracking function, checked for papers that had cited the papers included in our study but found none. We think that the relatively low number of papers on this topic suggests that there are simply very few empirical studies which focus on equity within international collaborations – a surprising finding especially considering the growth in collaborative practices in global health. The second limitation of this study is that we did not include normative accounts or other literature focussing on providing framework or guidelines related to equity in international collaboration and this is an equally important analysis that needs to be conducted going forward. The third limitation of this study is that our search was limited to studies in English, and may therefore have missed studies published in other languages.

## Conclusion

International collaborations have increasingly become a defining feature of global health research. While international collaborations offer important benefits, they have also been described as re-inscribing unequal power relations in favour of researchers and institutions situated in the global North. While there have been several attempts in the literature to address equity within international collaborations, to date, there had not been any attempts to summarise this evidence. Our scoping review, mapping evidence from qualitative studies and opinion and editorial pieces, has articulated 10 dimensions of equity. We argue that these 10 dimensions form the key areas which must be considered when developing equitable international collaborations.

## Data Availability

Not applicable

## Appendix

### Search strategy

**PubMed:**

Research Personnel [MeSH].

OR

Researcher OR researchers OR investigators OR scientists.

AND

International Cooperation [MeSH].

OR

Cooperative Behavior [MeSH].

OR

Collaboration OR collaborators OR cooperation OR international collaboration OR decision making OR international scientific collaboration OR international research collaboration.

AND

Social Justice [MeSH].

Social Responsibility [MeSH].

Ethics OR ethical OR equity OR equitable OR equality OR fair OR fairness OR values OR justice OR “social justice”.

AND

Empirical research [Mesh].

“Empirical studies” OR “qualitative research” OR “qualitative methods” OR opinion* OR perspective*.

**Scopus:**

Researcher OR investigator OR scientist.

AND

Collaboration OR collaborators OR cooperation OR (international collaboration) OR (decision.

making) OR (international scientific collaboration) OR (international research collaboration).

AND

Ethics OR ethical OR equity OR equitable OR equality OR fair OR fairness OR values OR justice OR “social justice”.

AND

“Empirical studies” OR “qualitative research” OR “qualitative methods” OR opinion OR perspective.

AND

**Web of Science:**

Researcher OR investigator OR scientist.

AND

Collaboration OR collaborators OR cooperation OR international collaboration OR decision making OR international scientific collaboration OR international research collaboration.

AND

Ethics OR ethical OR equity OR equitable OR equality OR fair OR fairness OR values OR justice OR “social justice”.

AND

“Empirical studies” OR “qualitative research” OR “qualitative methods” OR opinion OR perspective.

**EBSCO Host:**

Researcher OR investigator OR scientist.

AND

Collaboration OR collaborators OR cooperation OR “international collaboration” OR “decision making” OR “international scientific collaboration” OR “international research collaboration”.

AND

Ethics OR ethical OR equity OR equitable OR equality OR fair OR fairness OR values OR justice OR “social justice”.

AND

“Empirical studies” OR “qualitative research” OR “qualitative methods” OR opinion OR perspective.

## References

[1] Parker M, Kingori P. Good and bad research collaborations: Researchers’ views on science and ethics in global health research. PLoS One. 2016;11(10):e0163579. 10.1371/journal.pone.0163579. 10.1371/journal.pone.0163579 PMC5063577 27737006

[2] Matenga TFL, Zulu JM, Corbin JH, Mweemba O. Contemporary issues in north-south health research partnerships: Perspectives of health research stakeholders in Zambia. Health Res Policy Syst. 2019;17(1):7. 10.1186/s12961-018-0409-7. 10.1186/s12961-018-0409-7 PMC6334387 30646902

[3] Munung NS, Mayosi BM, De Vries J. Equity in international health research collaborations in Africa: Perceptions and expectations of African researchers. PLoS One. 2017;12(10):e0186237. 10.1371/journal.pone.0186237. 10.1371/journal.pone.0186237 PMC5643046 29036174

[4] Godoy-Ruiz P, Cole DC, Lenters L, McKenzie K (2016). Developing collaborative approaches to international research: perspectives of new global health researchers. Global Public Health..

[5] Ward CL, Shaw D, Anane-Sarpong E, Sankoh O, Tanner M, Elger B (2018). Defining health research for development: the perspective of stakeholders from an international health research partnership in Ghana and Tanzania. Dev World Bioethics.

[6] Hanney SR, González Block MA (2006). Building health research systems to achieve better health. Health Res Policy Syst.

[7] Airhihenbuwa CO, Shisana O, Zungu N, BeLue R, Makofani DM, Shefer T (2011). Research capacity building: a US-south African partnership. Glob Health Promot.

[8] Maziak W, Ward KD, Eissenberg T, Klesges RC, Keil U (2004). The Syrian Center for Tobacco Studies: a model of international partnership for the creation of sustainable research capacity in developing countries. Promot Educ.

[9] Van den Broucke S, Jooste H, Tlali M, Moodley V, Van Zyl G, Nyamwaya D (2010). Strengthening the capacity for health promotion in South Africa through international collaboration. Glob Health Promot.

[10] Yarmoshuk AN, Cole DC, Guantai AN, Mwangu M, Zarowsky C. The international partner universities of East African health professional programmes: Why do they do it and what do they value? Glob Health. 2019;15(1):37. 10.1186/s12992-019-0477-7. 10.1186/s12992-019-0477-7 PMC6555909 31174554

[11] Horton D, Prain G, Thiele G. Perspectives on partnership: a literature review. Int Potato Center. 2009;111:1–122.

[12] Bradley M. North-south research partnerships: challenges, responses and trends—a literature review and annotated bibliography. Working paper 1, IDRC Canadian Partnerships Working Paper Series Ottawa. Int Dev Res Centre. 2007.

[13] Crane J (2011). Scrambling for Africa? Universities and global health. Lancet.

[14] Crane JT (2013). Scrambling for Africa : AIDS, expertise, and the rise of american global health science.

[15] Boshoff N (2009). Neo-colonialism and research collaboration in Central Africa. Scientometrics..

[16] Costello A, Zumla A. Moving to research partnerships in developing countries. BMJ (Clin Res ed). 2000;321(7264):827–829. doi:10.1136/bmj.321.7264.827 BMJ. 10.1136/bmj.321.7264.827 PMC1118627 11009530

[17] Okeke IN (2016). African biomedical scientists and the promises of “big science”. Can J Afr Stud.

[18] Ruger JP (2009). Global health justice. Public Health Ethics.

[19] Pratt B, Hyder AA (2016). Governance of transnational global health research consortia and health equity. Am J Bioeth.

[20] Powers M, Faden RR (2019). Structural injustice : power, advantage, and human rights.

[21] Young IM. Justice and the politics of difference. New Jersey: New Jersey Princeton University Press; 1990.

[22] Maldonado-Torres N (2007). On the coloniality of being. Cult Stud.

[23] Fraser N, Peterson GB (1998). Social justice in the age of identity politics: redistribution, recognition, and participation. The Tanner lectures on human values.

[24] Fraser N (2000). Rethinking recognition. New Left Rev.

[25] Benhabib S. Toward a deliberative model of democratic legitimacy. In: Benhabib S, editor. Democracy and difference: contesting the boundaries of the political. New Jersey: New Jersey Princeton University Press; 1996.

[26] Fraser N (1997). Justice interruptus : critical reflections on the “postsocialist” condition.

[27] Fricker M (2011). Epistemic injustice : power and the ethics of knowing.

[28] Santos BS (2014). Epistemologies of the south: justice against epistemicide.

[29] Young IM. Inclusion and democracy. New York: New York Oxford University Press; 2000.

[30] Gould CC (2014). Interactive democracy : the social roots of global justice.

[31] Gutmann A, Thompson D. Why deliberative democracy? New Jersey Princeton University Press; 2010.

[32] Daniels N (2012). Just health: meeting health needs fairly.

[33] Ruger JP (2011). Shared health governance. Am J Bioethics.

[34] Powers M, Faden RR. Social justice : the moral foundations of public health and health policy. 2008. 10.1007/s00103-008-0443-7 18259708

[35] Nussbaum MC. Women and human development the capabilities approach. Cambridge: Cambridge Cambridge University Press; 2013.

[36] Venkatapuram S (2013). Health justice: an argument from the capabilities approach.

[37] Wolff J, De-Shalit A (2013). Disadvantage.

[38] Ruger JP (2010). Health and social justice.

[39] London AJ (2005). Justice and the human development approach to international research. Hastings Cent Rep.

[40] Pratt B, Hyder AA (2015). Global justice and health systems research in low- and middle-income countries. J Law Med Ethics.

[41] Pratt B, Wild V, Barasa E, Kamuya D, Gilson L, Hendl T (2020). Justice: a key consideration in health policy and systems research ethics. BMJ Glob Health.

[42] Millum J, Emanuel EJ. Global justice and bioethics. New York: New York Oxford University Press; 2015.

[43] Benatar SR, Singer PA (2010). Responsibilities in international research: a new look revisited. J Med Ethics.

[44] Slack C, Stobie M, Milford C, Lindegger G, Wassenaar D, Strode A (2005). Provision of HIV treatment in HIV preventive vaccine trials: a developing country perspective. Soc Sci Med.

[45] Bass E. Ethics, antiretrovirals and prevention trials. 2003 Contract No.: 3.

[46] Krubiner CB, Hyder AA (2014). A bioethical framework for health systems activity: a conceptual exploration applying ‘systems thinking’. Health Systems.

[47] Montreal Statement on Research Integrity in Cross-Boundary Research Collaborations. World Conferences on Research Integrity; 2013.

[48] Guidelines for research in partnership with developing countries. Commission for Research Partnership with Developing Countries (KFPE). 1988.

[49] The COHRED Fairness Index for international collaborative partnerships. 2015. 10.1016/S0140-6736(15)60680-8 25890912

[50] Morrison K, Tomsons S, Gomez A, Forde M (2018). Network of ethical relationships model for global north–south population health research. Glob Public Health..

[51] Munn Z, Peters MDJ, Stern C, Tufanaru C, McArthur A, Aromataris E (2018). Systematic review or scoping review? Guidance for authors when choosing between a systematic or scoping review approach. BMC Med Res Methodol.

[52] Arksey H, O'Malley L (2005). Scoping studies: towards a methodological framework. Int J Soc Res Methodol.

[53] Cacchione PZ (2016). The evolving methodology of scoping reviews. Clin Nurs Res.

[54] Braun V, Clarke V (2006). Using thematic analysis in psychology. Qual Res Psychol.

[55] Walsh A, Brugha R, Byrne E (2016). “The way the country has been carved up by researchers”: ethics and power in north-south public health research. Int J Equity Health.

[56] Parker M, Kingori P (2016). Good and bad research collaborations: researchers’ views on science and ethics in Global Health research. PLoS One.

[57] Moyi Okwaro F, Geissler PW (2015). In/dependent collaborations: perceptions and experiences of African scientists in transnational HIV research. Med Anthropol Q.

[58] Guzmán JAC, Espinal R, Báez J, Melgen RE, Rosario PAP, Mendoza ER (2017). Ethical challenges for international collaborative research partnerships in the context of the Zika outbreak in the Dominican Republic: a qualitative case study. Health Res Policy Syst.

[59] Muldoon KA, Birungi J, Berry NS, Ngolobe MH, Mwesigwa R, Shannon K (2012). Supporting southern-led research: implications for north-south research partnerships. Can J Public Health.

[60] Tindana P, Molyneux CS, Bull S, Parker M. Ethical issues in the export, storage and reuse of human biological samples in biomedical research: perspectives of key stakeholders in Ghana and Kenya. BMC Med Ethics. 2014;15(1):76. 10.1186/1472-6939-15-76. 10.1186/1472-6939-15-76 PMC4210627 25326753

[61] Binka F (2005). Editorial: north–south research collaborations: a move towards a true partnership?. Tropical Med Int Health.

[62] de Vries J, Munung SN, Matimba A, McCurdy S, Oukem-Boyer OOM, Staunton C, et al. Regulation of genomic and biobanking research in Africa: a content analysis of ethics guidelines, policies and procedures from 22 African countries. BMC Med Ethics. 2017;18:8. 10.1186/s12910-016-0165-6. 10.1186/s12910-016-0165-6 PMC5289015 28153006

[63] Jentsch B, Pilley C (2003). Research relationships between the south and the north: Cinderella and the ugly sisters?. Soc Sci Med.

[64] Moodley K, Singh S (2016). “It’s all about trust”: reflections of researchers on the complexity and controversy surrounding biobanking in South Africa. BMC Med Ethics..

[65] Metz T, Boisen C, Murray MC (2017). An African theory of social justice: relationship as the ground of rights, resources and recognition. Distributive justice debates in political and social thought.

[66] Pratt B, de Vries J (2018). Community engagement in global health research that advances health equity. Bioethics..

[67] Pierson L, Millum J (2018). Health research priority setting: the duties of individual funders. Am J Bioeth.

[68] Pratt B, Hyder AA (2018). Priority setting is more than resource allocation: reflecting on the content of funders’ duties and their implications for current practice. Am J Bioeth.

[69] de Vries J, Tindana P, Littler K, Ramsay M, Rotimi C, Abayomi A (2015). The H3Africa policy framework: negotiating fairness in genomics. Trends Genet.

[70] IJsselmuiden C, Marais DL, Becerra-Posada F, Ghannem H (2012). Africa’s neglected area of human resources for health research – the way forward. S Afr Med J.

[71] Lansang MA, Dennis R (2014). Building capacity in health research in the developing world. Bull World Health Org.

[72] Wight D (2008). Most of our social scientists are not institution based … they are there for hire—research consultancies and social science capacity for health research in East Africa. Soc Sci Med.

[73] Crane JT, Andia Biraro I, Fouad TM, Boum Y (2018). R. Bangsberg D. the’ indirect costs’ of underfunding foreign partners in global health research: a case study. Glob Public Health.

[74] Adedokun BO, Olopade CO, Olopade OI (2016). Building local capacity for genomics research in Africa: recommendations from analysis of publications in sub-Saharan Africa from 2004 to 2013. Glob Health Action.

[75] Hedt-Gauthier BL, Jeufack HM, Neufeld NH, Alem A, Sauer S, Odhiambo J (2019). Stuck in the middle: a systematic review of authorship in collaborative health research in Africa, 2014–2016. BMJ Glob Health.

[76] Rees CA, Lukolyo H, Keating EM, Dearden KA, Luboga SA, Schutze GE (2017). Authorship in paediatric research conducted in low- and middle-income countries: parity or parasitism?. Trop Med Int Health.

[77] Pingray V, Ortega V, Yaya S, Belizán JM (2020). Authorship in studies conducted in low-and-middle income countries and published by reproductive health: advancing equitable global health research collaborations. Reprod Health.

[78] Crane JT (2020). Dreaming partnership, enabling inequality: administrative infrastructure in global health science. Africa..

[79] Peters MDJ, Godfrey CM, Khalil H, McInerney P, Parker D, Soares CB (2015). Guidance for conducting systematic scoping reviews. Int J Evid Based Healthcare.

